# Ambulance Nurses' Competence and Perception of Competence in Prehospital Trauma Care

**DOI:** 10.1155/2018/5910342

**Published:** 2018-04-23

**Authors:** Anna Abelsson, Lillemor Lindwall, Björn-Ove Suserud, Ingrid Rystedt

**Affiliations:** ^1^Department of Nursing Science, School of Health Sciences, Jönköping University, P.O. Box 1026, 551 11 Jönköping, Sweden; ^2^Department of Health Sciences, Karlstad University, Karlstad, Sweden; ^3^Faculty of Caring Science, Work Life and Social Welfare, PreHospen-Centre for Prehospital Research, University of Borås, Borås, Sweden

## Abstract

**Introduction:**

We focus on trauma care conducted in the context of a simulated traumatic event. This is in this study defined as a four-meter fall onto a hard surface, resulting in severe injuries to extremities in the form of bilateral open femur fractures, an open tibia fracture, and a closed pelvic fracture, all fractures bleeding extensively.

**Methods:**

The simulated trauma care competence of 63 ambulance nurses in prehospital emergency care was quantitatively evaluated along with their perception of their sufficiency. Data was collected by means of simulated trauma care and a questionnaire.

**Results:**

Life-saving interventions were not consistently performed. Time to perform interventions could be considered long due to the life-threatening situation. In comparison, the ambulance nurses' perception of the sufficiency of their theoretical and practical knowledge and skills for trauma care scored high. In contrast, the perception of having sufficient ethical training for trauma care scored low.

**Discussion:**

This study suggests there is no guarantee that the ambulance nurses' perception of theoretical and practical knowledge and skill level corresponds with their performed knowledge and skill. The ambulance nurses rated themselves having sufficient theoretical and practical knowledge and skills while the score of trauma care can be considered quite low.

## 1. Introduction

Prehospital trauma care situations are rarely ideal conditions for providing care [1]. The complexity of the situation requires well-trained ambulance staff [2–4]. Caring for a patient that has sustained a traumatic injury involves knowledge of the actual processes that the patient is currently experiencing but also knowledge of the potential injury that has yet to make itself known [5]. Previous research describes the competence needed in prehospital emergency care, for example, intubation, trauma care, CPR, ventilation, and triage [6–8]. Simulation is an educational method that is increasingly used where the ambulance nurses in prehospital emergency care practice can improve their knowledge, skills, and experience in the care of injured patients [9]. Simulation makes it possible for ambulance nurses to participate in realistic and complex situations. The learning takes place through continuous learning and includes reflection over the simulated experience [10–12]. Prior research has described the simulated learning of different care actions, such as external compression to temporize exsanguination [13] or the difficulties with intubating a prehospital patient [14, 15].

The Swedish National Board of Health and Welfare [16] points out that all prehospital personnel should have adequate trauma training to ensure a good quality trauma care. The educational background of staff in Swedish ambulances is varied: emergency medical technicians with vocational training and registered nurses with a three-year Bachelor degree at the university level. There are also registered nurses with an additional one-year national specialist education at university level, including a master thesis. These specialist educations are professional titles and include specialist ambulance nurse, specialist intensive care nurse, or specialist anaesthesia nurse.

The aim of this study was to examine actual individual trauma care competence among ambulance nurses in prehospital emergency care practice. One additional aim was to examine the individual perceptions of trauma care knowledge and skills, experience, and training.

## 2. Methods

This study has a quantitative design with a cross-sectional descriptive design, conducted in the context of a simulation exercise. We evaluated the simulated trauma care competence of 63 ambulance nurses in prehospital emergency care and in relation to the simulation collected data, by means of a questionnaire, on their perception of having sufficient trauma care knowledge and skills, experience, and training.

### 2.1. Sample

In total, 63 ambulance nurses participated in the study: 29 females and 37 males, aged between 25–63 years (mean 40). The participants were registered nurses (*n* = 23) and specialist nurses (*n* = 30). In this study, all nurses working in the ambulance are hereafter referred to as ambulance nurses. The participants worked in prehospital emergency care practice in five different regions in Sweden. The mean experience from prehospital emergency care was 9 years (range 1–30 years). All participants were informed about the study by their station chief and the nurses who were willing to participate contacted the researcher themselves. Inclusion criteria were ambulance nurses in prehospital emergency care participating in a simulation. All participants had previous experience of simulation.

### 2.2. Setting

The study took place on the garage floor in the participants' respective ambulance stations. The scenarios were carried out by the participants in pairs, based on which participants were on duty at the time. They were randomly assigned the primary or secondary role in the simulation. The primary ambulance nurse was instructed to assess and perform trauma care as if at a real accident site. The secondary ambulance nurse was asked not to take any independent actions, nor make suggestions for trauma care. The simulation was performed twice per pair and for each simulation, the focus was on the primary ambulance nurse.

The participants received a fictitious call from dispatch regarding a patient who had fallen four meters onto the ground. Any other information about the patient's situation was unknown. A person who had witnessed the accident, impersonated by the facilitator, met the participants at the scene of the simulated accident. They were then shown a picture of the scene of the accident. A Resusci Anne Basic manikin (Laerdal®) represented a patient with an obstructed airway and was moulaged with severe injuries to extremities in the form of bilateral open femur fractures, an open tibia fracture, and a closed pelvic fracture, all bleeding extensively. The scenarios were constructed with two specialists in prehospital trauma care to ensure content validity.

The trauma care was based on the Prehospital Trauma Life Support (PHTLS®) mnemonic concept Airway, Breathing, Circulation, Disability, and Exposure, ABCDE [5], used in the clinical prehospital practice by all ambulance organizations in Sweden [16]. Vital parameters were given by the facilitator during the scenario. The facilitator also adjusted the patient outcome based on the interventions made during the scenario [17]. The participants did not receive any feedback during the scenarios. The scenario ended when the primary ambulance nurse stated that they were ready to leave the scene. The scenario was followed by a debriefing led by the facilitator. Correct actions were confirmed while wrong or overlooked care actions were highlighted and theoretically corrected.

All scenarios were filmed because these simulated emergency situations were considered impossible to rate appropriately in real-time. No other persons were present during the simulation. The participants were in no way dependent on the researcher and were informed about the aim of the research before the simulation.

### 2.3. Rating Scale

For evaluation of the participants' trauma care knowledge and skills, the Global Rating Scale for the assessment of paramedic clinical competence (GRS), validated on paramedics in prehospital emergency practice, was used (Table 1) [18]. Consent to use the GRS was obtained from the developer.

The raters scored the GRS seven dimensions on a 7-point Likert scale. All seven dimensions were calculated for a total score representing the overall clinical performance [18]. A total GRS score of 7–21 was regarded unsafe/weak, a total GRS score of 22–28 was marginal, and a total GRS score of 29–49 was competent.

A supplementary flowchart, based on the concept ABCDE [5], including the care actions expected by the ambulance nurses in prehospital emergency care during care of a trauma patient was used to enable a more detailed evaluation of the participants' care actions in the dimension of* patient assessment*. The flowchart consisted of criteria (examinations, assessments, and care actions) that the participants were expected to perform according to the PHTLS concept. For each criterion, the contents of the supplementary flowchart were marked by raters with “yes” or “no.” Before the start of the study, certain criteria were classified as critical criteria. The time taken before these criteria were performed was measured. The supplementary flowchart was constructed based on current research, and, additionally, with the input from two specialists in prehospital trauma care to ensure content validity.

### 2.4. Raters of the GRS and the Flowchart

The two raters were prehospital emergency personnel with an educational function within their respective organizations. They had previous experience of evaluation of simulation in a prehospital emergency context. The raters had no previous knowledge about the participants and they made their assessments independently. They were, prior to the study, educated in the use of GRS and the flowchart and provided with information on the patient scenario. To test the interrater reliability [19], the raters initially evaluated the same 14 video-recorded scenarios. The interrater reliability was 95% with 5 differences in 98 items. The remaining recordings were then randomly divided between the two and evaluated separately.

### 2.5. Questionnaire

A questionnaire consisting of 9 questions was used to assess the participants' self-rated perception of having sufficient level of trauma care provision. The questionnaire covered the areas of theoretical/practical/ethical knowledge and skills/experience/training of trauma care in prehospital emergency care. The questions were asked in the form of assertions that the participants marked on a 5-point Likert scale. The rating of the Likert scale ranged from 1 = disagree to 5 = agrees completely. The questions aimed at answering participants' self-rated perception of knowledge and skills, experience, and training in the areas of medical theory, practical nursing, and ethics. The questionnaire was developed with input from two experts in education and trauma care to ensure content validity. The participants answered the questionnaire after performing the simulation.

### 2.6. Data Analysis

Descriptive statistics were produced using IBM Statistical Package for the Social Sciences (SPSS) 24.0. Descriptive analysis, such as central tendencies and distributions, were used to describe the data.

*Ethical Consideration*. The study followed the ethical principles of the World Medical Association [20] in regard to anonymity and integrity. Ethical approval for this study was obtained from the Institutional Review Board. Informed consent was obtained from each participant. No unauthorized person has had access to the material.

## 3. Result

The result presents the actual trauma care competence, and the evaluative measures indicated that some assessments, examinations, and care actions were not consistently performed, even those actions that could be considered life-saving interventions. The care actions in the result are not rated as right or wrong, just performed or not.

Table 2 describes the proportion of participants performing a number of care actions during the simulated trauma care. Examination of the patient's head was conducted by 13% of the participants, while 16% assessed the patient's level of consciousness. Inspection of the patient's mouth was conducted by 21% of the participants and assessment of the patient's pupil reactions was conducted by 25%. The care action of exposure was conducted by 31% of the participants. In comparison, 93% of the participants did, however, apply a cervical collar on the patient (Table 2).

As seen in Table 3, the time before performing a jaw thrust was long. The performance of a jaw thrust was classified as a critical criterion, and three participants never performed it. Stopping the extensive bleeding from the legs was also a critical criterion due to the life-threatening danger it posed. Regardless, eight participants neither identified nor stopped the bleeding (Table 3).

Table 4 shows the scores for the seven dimensions of GRS on a 7-point Likert scale. For all seven dimensions, no participant reached the score of 7. It is noteworthy that the individually lowest total score was 10 which was considered unsafe/weak (Table 4).

Regarding the ambulance nurses' self-rated perceptions of having sufficient trauma care knowledge and skills, Table 5 shows that the self-rated perception of theoretical training, practical training, and ethical experiences for working in a prehospital emergency practice scored low. In comparison, the self-rated perception of having sufficient theoretical knowledge and skills scored the highest of all items, followed by the second highest scored item, practical knowledge, and skills. The self-rated perception of having sufficient ethical training for a prehospital emergency practice scored the lowest of all items (Table 5).

## 4. Discussion

The result in the study showed that the ambulance nurses did not feel like they had enough training (theoretical 45%, practical 50%) or experience (theoretical 63%, practical 65%) regarding trauma care. It showed in their lack of ability to provide adequate care in a simulated trauma care practice (patient assessment 57%, a total score of all dimensions 63%). During the simulation, the ambulance nurses did not always perform essential assessments or vital care actions needed to save the patient's life at the scene of the accident. A detailed examination of the flowchart revealed that it took a long time before a jaw thrust was performed, which is a life-saving intervention. Assistance with an open airway and thereby functioning ventilation is paramount to the welfare of patients exposed to high energy trauma who are not able to maintain an open airway themselves [21].

In addition, it took a long time to stop the bleeding from the bilateral open fractures in the legs, based on the severity of the situation. Early detection of bleeding is a prerequisite for rapid action, so that blood loss is minimized, and the patient's vital signs are stabilized [22].

It is noteworthy that the examination of the patient's head and the assessment of pupil reactions were seldom conducted during the simulation, meaning that possible traumatic brain injuries were not identified. This can be life-threatening for the patient since patients suffering from severe trauma often also suffer from traumatic brain injuries [23]. This, in turn, can have a major impact on the overall survival of the patient.

The ambulance nurse's self-rated perceptions of having sufficient theoretical knowledge and skills (71%) in conjunction with having sufficient practical knowledge and skills (70%) for trauma care were, in our study, the highest rated items. Our result is confirmed by other studies showing the increased confidence that the participants get after simulation [24–26]. However, when comparing the participants' self-rated perception of having sufficient theoretical knowledge and skills and practical knowledge and skills for trauma care to the actual competence observed during the simulation exercise (patient assessment 57%), the two results suggest a discrepancy. Previous research has shown, in contrary to this study where the mean experience as a nurse was 9 years, how the discrepancy decreases as the nurses' competence levels improve as they acquire more clinical experience [27]. The discrepancy in perceived and actual competence will differ rendering different clinical experience and length of clinical experience [28].

The self-rated perception of having sufficient theoretical trauma care training (45%) and practical training (50%) scored low, implying that the ambulance nurses in prehospital emergency care feel the need for more training. This becomes an interesting contradiction in the result as the ambulance nurses scored the self-rated perception of having sufficient theoretical and practical knowledge and skills high (71%/70%). This need for more training has previously been identified in military emergency care contexts [29]. Axelsson et al. [30] describe that the opportunity for learning trauma care in prehospital emergency care is during the clinical placement in ambulance nurse students' education. Though, most ambulance districts offer further education and training.

The self-rated perception of having sufficient ethical training for trauma care was rated the lowest of all items in this study (39%). This is worrisome because moral distress among ambulance nurses has previously been identified when ethical support is too low [31–33]. When nurses can reflect on their own feelings, it enables them to feel compassion for the patient. The nurse can then act in a constructive manner based on the acute situation and with the best interest of the patient in hand [34].

## 5. Limitations

In this study, one limitation may be that ambulance nurses interested in trauma and trauma care simulation were more likely to participate in the study which could have affected the simulated outcome. Another limitation may be that the simulation was performed twice per pair which gave the secondary ambulance nurse an advantage in the scenario since they were given more time to prepare and reflect. It should be noted that neither the primary nor the secondary ambulance nurse rarely realized that it was the same scenario twice. A limitation of using a self-rated questioner may be the participants' varied capacity of introspective ability. Even in an attempt to be honest, the lack of introspective ability can result in the participants being unable to judge themselves correctly [35].

### 5.1. Implications for Ambulance Nurses in Prehospital Emergency Care

Education and training resulting in knowledge and experience is essential for ambulance nurses in prehospital emergency care. This is especially true in acute and life-threatening trauma situations where decision-making and care actions are performed under time pressure. This research shows a discrepancy between the observed trauma care competence and the perception of having sufficient trauma care competence, which can potentially cause a false sense of competence. However, it appears that actual competence still needs to be evaluated. The improved self-confidence that simulation of trauma care gives rise to is likely an important factor in the care of a critical patient. However, it is also important that the actual trauma care competence is well aligned with the level of self-confidence.

## 6. Conclusion

The results suggest that the participants in the context of a simulation exercise rate themselves as having sufficient theoretical and practical knowledge and skills. However, the evaluation of the performed assessment, examinations, and care actions shows that crucial care actions are not always performed. The simulation may provide participants with improved self-confidence, but there is no guarantee that this confidence is well aligned with the actual competence.

## Figures and Tables

**Table 1:** The GRS seven dimensions and the scoring on the 7-point Likert scale.

The GRS seven dimensions						
Situation awareness		The ability to consider the environment, anticipate possible events, avoid inappropriate tunnel vision, and continuously update actions when necessary.				
History gathering		Gather and organize structured, timely, and focused medical history according to the clinical situation and context.				
Patient assessment		Appropriate physical examination according to clinical status and level of urgency.				
Decision-making		Well-informed decisions made based on appropriate prioritizing.				
Resource utilization		Identify and use resources efficiently to maximize care.				
Communication		The ability to communicate (verbally, listening, closing the loop, and body language) with the team, patients, and bystanders.				
Procedural skill		Level of psychomotor skills and adaptability to problems.				

The 7-point Likert scale						

(1) Unsafe	(2) Unsatisfactory	(3) Poor/weak	(4) Marginal	(5) Competent	(6) Highly competent	(7) Exceptional

**Table 2:** The proportion of participants who performed specific assessments and care actions during the simulation.

Care actions	%
Applied cervical collar	93%
Talked to the patient upon arrival	88%
Spinal immobilization	86%
Stabilized the cervical spine by hand	84%
Followed the ABCDE algorithm	71%
Oropharyngeal airway	69%
Inspection of chest	68%
Assessed respiratory rate	68%
Examination pelvis	66%
Examination abdomen	55%
Examination thorax	54%
Breathing sounds	49%
Assessed airway management	44%
Exposure	31%
Pupil reactions	25%
Opened mouth for inspection	21%
Assessed level of consciousness	16%
Examination head	13%

**Table 3:** The response time to critical care actions.

Care actions	Mean (seconds)
Jaw thrust mean	35
SD	(37)
Min time–max time	5–240
Undertook no action	5% (*n* = 3)

Radial pulse mean	83
SD	(69)
Min time–max time	17–525
Undertook no action	11% (*n* = 7)

Oxygen mask mean	114
SD	(81)
Min time–max time	3–375
Undertook no action	8% (*n* = 5)

Stop bleeding leg mean	202
SD	(105)
Min time–max time	31–470
Undertook no action	13% (*n* = 8)

Leaving the scene mean	395
SD	(130)
Min time–max time	164–660

**Table 4:** Score per dimension, total score on the 7-point Likert scale, and percentage of 7 points.

Dimension	Mean	Percentage of 7 points
Situation awareness	4.43	4.43 of 7 = 63%
SD	(.91)	
Min score–max score	2–6	

History gathering	4.06	4.06 of 7 = 58%
SD	(1.01)	
Min score–max score	2–6	

Patient assessment	4.02	4.02 of 7 = 57%
SD	(1.04)	
Min score–max score	1–6	

Decision-making	4.18	4.18 of 7 = 60%
SD	(1.1)	
Min score–max score	1–6	

Resource utilization	4.18	4.18 of 7 = 60%
SD	(.96)	
Min score–max score	1–6	

Communication	4.36	4.36 of 7 = 62%
SD	(.94)	
Min score–max score	2–6	

Procedural skill	4.07	4.07 of 7 = 58%
SD	(.97)	
Min score–max score	1–6	

Total score	29,49	29.49 of 7 = 63%
SD	(6,29)	
Min score–max score	10–42	

**Table 5:** Participants self-rated perception on the 5-point Likert scale rated mean in the questionnaire regarding *I have sufficient *theoretical/practical/ethical knowledge and skills/experience/training.

Item *I have sufficient*	Mean (SD)	Percentage of 5 points
Theoretical knowledge and skills for trauma care	3.54 (.82)	3.54 of 5 = 71%
Theoretical experience of trauma care	3.16 (.99)	3.16 of 5 = 63%
Theoretical training for trauma care	2.27 (.95)	2.27 of 5 = 45%
Practical knowledge and skills for trauma care	3.48 (.80)	3.48 of 5 = 70%
Practical experience of trauma care	3.27 (.86)	3.27 of 5 = 65%
Practical training for trauma care	2.48 (.95)	2.48 of 5 = 50%
Ethical knowledge and skills for trauma care	3.02 (.99)	3.02 of 5 = 60%
Ethical experience of trauma care	2.73 (1.10)	2.73 of 5 = 55%
Ethical training for trauma care	1.94 (.88)	1.94 of 5 = 39%
